# 云南省宣威市肺癌危险因素研究

**DOI:** 10.3779/j.issn.1009-3419.2017.08.05

**Published:** 2017-08-20

**Authors:** 利群 刘, 霞 万, 功博 陈, 祥云 马, 伯福 宁, 功焕 杨

**Affiliations:** 1 100730 北京，中国医学科学院基础医学研究所暨北京协和医学院基础学院 Institute of Basic Medical Sciences, Chinese Academy of Medical Sciences & School of Basic Medicine, Peking Union Medical College, Beijing 100730, China; 2 655400 宣威，云南省宣威市疾病预防控制中心 Xuanwei Center for Disease Control and Prevention, Xuanwei 655400, China

**Keywords:** 宣威, 肺肿瘤, 肺癌危险因素, 描述性统计, 暴露率, 标化率, Xuanwei, Lung neoplasms, Risk factors of lung cancer, Descriptive statistics, Exposure percentage, Standardized rate

## Abstract

**背景与目的:**

自1970年代至今，云南省宣威市始终是我国肺癌高发地区之一，且其女性高发、地区聚集性高发的特征未发生过改变。本研究目的在于进一步关注宣威市普通居民肺癌高发危险因素暴露的变化情况，以预测未来肺癌分布特征。

**方法:**

根据2010年-2012年肺癌死亡率将宣威市26个乡镇划分为高、中、低发区后，根据地形和方位选取6个乡镇（高中低发区各2个），并从每个乡镇中随机抽取4个行政村作为调查点。对调查点中居民样本人群进行肺癌危险因素面对面调查。计算高、中、低发区人群危险因素暴露率，以及性别、年龄别亚组人群暴露率，其中生活环境方面危险因素计算现在和十年前。采用标化率和标化率比较的统计学检验或卡方检验，比较区域间或两时间点暴露水平的高低。

**结果:**

研究地区65%-80%的男性有吸烟史，60%-90%的从未吸烟者有二手烟暴露史。烟草使用和二手烟暴露均是在肺癌高、中发区明显严重于低发区。中发区50%的男性有煤矿工作经历，是高、低发区的2倍；而高发区15%-25%的人有其他职业烟尘暴露史，是中、低发区的3倍-5倍。从十年前到现在，中发区都有近80%的家庭每年使用2吨或更多烟煤，有超90%的家庭使用燃煤取暖，有60%多的家庭做饭时厨房油烟多，有60%的家庭日常最常使用有烟囱地炉。高发区目前倒只有20%的家庭大量使用烟煤。整个研究地区50%-75%的家庭每年使用700度或更多电，比十年前用电量大增。低发区80%的人经常吃高油脂或腌制熏制食品，高、中发区为50%-60%。

**结论:**

从本次调查数据来看，目前烟煤使用情况的分布与肺癌高中低发区的分布已不一致。烟草流行包括二手烟暴露，烟煤使用，职业暴露可能是当前宣威肺癌仍旧高发的原因。炉灶类型、饮食习惯等危险因素与宣威肺癌间的相关性值得进一步研究。

## Abstract (en)

**Background and objective:**

Since 1970s, Xuanwei in Yunnan province has been one of the towns with highest lung cancer mortality in China. Moreover, the characters of high female lung cancer mortality and subregional clustering high lung cancer mortality have not changed. In this study, we further described the exposure situation of risk factors of lung cancer in Xuanwei nowadays, in order to explore the trend of the distribution of lung cancer there.

**Methods:**

Firstly we divided the 26 towns of Xuanwei city to high-, median-and low-lung cancer areas by the lung cancer mortality in 2010-2012. We chose 2 towns within each area according to topography and orientation, and randomly picked 4 villages in each town to be our study area. We did a questionnaire about lung cancer related risk factors upon the sample population in the study area. We calculated the exposure percentages of each risk factor, in whole sample population and subgroups, for nowadays and for 10 years ago (only living environmental risk factors), and compared them between areas or time points using standardized rates and the statistical test of standardized rate comparison, or chi-square test.

**Results:**

65%-80% male in the study area has a history of smoking; 60%-90% nonsmoker has been exposed to second hand smoke. These situations are worse in high and median lung cancer areas. 50% male in median lung cancer area have coal mining work experience, which is 2 times of the percentages in the other two areas; while 15%-25% people in high lung cancer area have other occupational exposure history to particulate air pollution, which is 3-5 times of the percentages in the other two areas. From ten years ago until nowadays, 80% families in median lung cancer area use 2 tons or more smoky coal per year; more than 90% families burn coal for household heating; more than 60% families suffer from smog in the kitchen during cook; 60% families most frequently use stove in the ground with chimney. Only 20% families in high lung cancer area now use 2 tons or more smoky coal per year. Now 50%-75% families in the study area use 700 kilowatt-hours of electricity per year, much more than ten years ago. 80% residents in low lung cancer area eat fatty or pickled or smoked food at least 3 days per week; while in high and median lung cancer areas the percentages are 50%-60%.

**Conclusion:**

According to data obtained in this survey, current distribution of smoky coal use has differed from the distribution of high-, median-and low-lung cancer areas. Tobacco use and second hand smoke, the use of smoky coal and occupational exposure to particulate matters could be the main risk factors for lung cancer in Xuanwei now. The relations between lung cancer and stove type, dietary habit and so on deserve further study.

云南省宣威市是我国肺癌死亡率最高的地区之一。1973年-1975年开展的全国恶性肿瘤死亡回顾调查数据显示，宣威市男性和女性肺癌死亡率分别为27.7/10^5^和25.3/10^5^^[1]^，是同期全国男女肺癌死亡率的4.1倍和7.9倍。既往研究结果^[2, 3]^表明，利用无烟囱火塘燃烧烟煤造成的严重室内空气污染，是宣威地区肺癌的主要危险因素。因此，宣威肺癌具有高发、女性高发、地区聚集性高发的特征。基于前期研究结果，1980年代起至1995年宣威市进行了大规模的改炉改灶工程。调查和研究证明，工程结束后宣威市无烟囱火塘的使用率大幅下降，室内颗粒物和致癌物质苯并[a]芘的浓度下降，男女患肺癌的风险均显著下降^[4-6]^。然而，宣威市肺癌死亡率并未如预期的那样随之下降。比较1973年-1975年、1990年-1992年及2004年-2005年三次全国死因抽样调查数据可见，宣威肺癌死亡率是持续上升的，且与全国农村肺癌死亡率水平间的差距不断加大^[7]^。2011年-2013年宣威市死因登记数据经漏报调整后，与2004年-2005年相比，可见肺癌死亡率仅略有下降（5%），男性和女性肺癌死亡率仍分别为全国农村水平的3倍和6倍。为更加深入系统地研究影响宣威肺癌的因素，本课题组于2014年8月在宣威市开展了针对普通居民的肺癌危险因素调查。现通过描述宣威地区人群肺癌危险因素的暴露特征，来探索其疾病分布特征的成因。

## 1 对象和方法

### 1.1 调查点选择

调查点的选择分两步进行：第一步在收集常规死因登记数据、人口数据的基础上（具体细节可见^[7]^中“资料来源”部分），计算宣威市26个乡镇2010年-2012年的肺癌死亡率，依据其三分位数（100/10^5^和50/10^5^）将26个乡镇划分为肺癌高发区、中发区和低发区，再根据地形和方位，在每个区域选择两个乡镇。最终选中的为肺癌高发区的来宾镇、宛水街道，肺癌中发区的倘塘镇、格宜镇，肺癌低发区的热水镇、文兴乡。第二步在每个乡镇随机选择4个行政村作为调查点。全部调查点为6个乡镇的24个行政村。

### 1.2 现场调查

本次调查的调查员由各行政村的村医在经过培训后担任，调查过程全部在平板电脑上操作。调查员按照一定的原则选择起点和路线，并按照既定的规则沿每条路线进行入户调查。具体细节可参见^[7]^中对现场调查过程的说明和现场调查路线图。每个行政村入户100家。入户后，调查员先记录下所选家庭每一位成员的信息，包括姓名、性别、年龄、与户主关系、近两周是否在家等；再从30岁以上、近期在家的成员中随机选择一位作为调查对象。即每个行政村计划访问100名村民。

### 1.3 调查问卷

调查问卷由九部分内容组成：知情同意书、调查对象一般情况、职业史、现在和十年前的生活居住环境（包括燃料和炉灶的使用情况）、饮食模式、吸烟史、二手烟暴露情况、饮酒史、既往肺部疾病史和亲属肿瘤史。

### 1.4 质量控制和伦理

首先，本次调查采用了电子版问卷，形式为在平板电脑上操作的调查系统，在调查过程中，由调查系统自动完成问卷的逻辑控制和完整性核查。第二，所有调查员都经过培训合格后才开始投入工作。第三，调查员每天通过电子邮件将当天调查数据传送给本课题组工作人员，由工作人员审核数据质量并及时反馈问题。第四，本课题组工作人员每*1*至2周会到现场随同调查员一起访问，确保现场调查工作遵循既定的规则进行。本研究通过了北京协和医学院伦理审查委员会的审查。所有研究对象均签署了知情同意书。

### 1.5 统计分析

本研究首先对调查样本人群的特征进行了描述性统计。之后，计算肺癌高、中、低发区人群各个危险因素的暴露率。由于三个区的样本人群年龄结构显著不同（详见结果部分表 1），以直接法计算标化率后，在区域间比较暴露水平的高低，并做统计学检验。直接法选取2010年全国人口普查的年龄分布作为标准人口年龄结构。对于生活居住环境（包括燃料和炉灶的使用情况）方面的危险因素，还在各区内比较了现在和十年前暴露率水平的差异。此时的比较采用卡方检验，当四格表中有单元格的期望值小于5时采用Fisher精确检验。在完成对整个样本人群的分析后，将样本人群按照性别或年龄分成亚组，在亚组内重复上述描述性统计和比较过程。其中，在年龄别亚组中进行区域间暴露率水平的比较时，也采用卡方检验或*Fisher*精确检验。比较的显著性检验标准取*α*=0.05。全部统计分析由SPSS 22.0完成。

**1:** 调查对象人口学特征 Socio-demographic characteristics of the respondents

Characteristics	High lung cancer area			Median lung cancer area			Low lung cancer area		*χ*^2^	*P*
	*n*	%		*n*	%		*n*	%		
Gender									2.41	0.299
Male	325	43.7		366	46.4		348	42.7		
Female	418	56.3		422	53.6		467	57.3		
Age (yr)									21.99	0.001
30-39	187	25.2		254	32.2		237	29.1		
40-49	248	33.4		266	33.8		247	30.3		
50-59	187	25.2		176	22.3		182	22.3		
60-69	121	16.3		92	11.7		149	18.3		
Ethnic group									68.48	< 0.001
Han	703	94.6		782	99.2		732	89.8		
Minority	40	5.4		6	0.8		83	10.2		
Marriage									6.26	0.181
Married or living together	684	92.1		732	92.9		770	94.5		
Widowed	40	5.4		33	4.2		37	4.5		
Single or divorced	14	1.9		19	2.4		8	1.0		
Unknown	5	0.7		4	0.5					
Education									72.06	< 0.001
Junior high and above	307	41.3		169	21.4		239	29.3		
Primary school or illiterate	436	58.7		619	78.6		576	70.7		
Annual family income (Yuan)									49.94	< 0.001
≥20, 000	225	30.3		267	33.9		410	50.3		
< 20, 000	247	33.2		515	65.4		392	48.1		
Refuse to answer	271	36.5		6	0.8		13	1.6		
Occupation									290.36	< 0.001
Farmer	570	76.7		658	83.5		659	80.9		
Other	173	23.3		130	16.5		156	19.1		
Ever left Xuanwei									9.07	0.059
No	710	95.6		769	97.6		803	98.5		
Yes	15	2.0		19	2.4		5	0.6		
Unknown	18	2.4					7	0.9		
Age(yr)	Mean±SD	Median		Mean±SD	Median		Mean±SD	Median		
	47.71±10.25	47		45.67±9.93	44		47.32±10.88	46		
Chi-square and P value were the results of the comparison of the three areas.										

## 2 结果

### 2.1 调查对象特征

本次调查的样本人群分布在上述6个乡镇24个行政村内，总数为2, 346人，其中肺癌高发区743人，中发区778人，低发区815人。被调查者的人口学特征见表 1。

如表 1所示，肺癌高、中、低发区的研究对象性别比例基本为男性45%左右，女性55%左右。年龄分布为40岁-49岁组人数最多，其次是30岁-39岁组，这两个年龄组占总人数的60%-65%；50岁-59岁组占25%左右，60岁-69岁组人数最少；没有70岁及以上的受访者。中发区研究对象较高、低发区年轻化，30岁-39岁人群占比更高，60岁-69岁人群占比更低。生活在肺癌高、中、低发区的研究对象基本都未离开过宣威市，75%以上职业为农民。90%以上为汉族、已婚。中发区的研究对象中仅有21%接受过初中或以上水平的教育，其余近80%为小学文化程度或文盲；低发区初中或以上教育水平者为30%，高发区为41%。低发区的研究对象家庭年收入在2万元及以上的占50%，中发区和高发区均为30%左右，但高发区居民有1/3强烈拒绝告知其家庭年收入。

### 2.2 生活环境方面危险因素的暴露情况

如表 2所示，中发区以燃煤方式取暖的家庭最多，占比93%；高发区最少，占比53%。高发区半数居民家中有抽油烟机或做饭时会注意开窗通风，中、低发区能达到这一项的均不足30%；然而低发区只有不到40%的人反映自家做饭时厨房油烟多、经常感觉眼咽刺激，与高发区类似，两区均低于中发区的60%。中发区有76%的家庭年平均用煤量在2吨或以上，低发区降至54%，高发区又锐减至20%。中发区有近50%的家庭年平均用电量在700度或以上，高、低发区都达到75%。三区均只有不足2%的家庭日常最常使用无烟囱火塘。中发区有61%的家庭最常使用有烟囱地炉，远高于另外两区。低发区有28%的家庭最常使用手提炉，远高于另外两区。低发区有35%的家庭最常使用有烟囱高灶，高发区有26%，中发区有20%。联合上述四类炉灶使用的数据判断，大多数（65%）高发区家庭最常使用的应为电炉。由于电炉不需燃烧烟煤，不会释放烟尘，暂未将其包含在表 2内。

**2:** 调查对象生活环境方面危险因素当前暴露情况 The exposure to living environmental risk factors of the respondents

Factors		High lung cancer area (*n* =743)	Median lung cancer area (*n* =788)	Low lung cancer area (*n* =815)	High lung cancer area			Median lung cancer area			Low lung cancer area	
					Standardized rate	95%CI		Standardized rate	95%CI		Standardized rate	95%CI
Ways of domestic heating involving smoky coal combustion^a^	Yes (*vs* No)	53.30%	93.27%	80.98%	52.99%	49.38%-56.60%		93.28%	91.52%-95.03%		81.01%	78.31%-83.71%
Ventilation or using smoke exhauster during cooking	Yes(*vs* No)	52.36%	26.90%	27.98%	52.57%	48.95%-56.18%		26.84%	23.75%-29.94%		27.56%	24.50%-30.62%
Fume in the kitchen during cooking	Thick (*vs* Thin)	36.61%	58.76%	38.04%	36.14%	32.68% -39.59%		58.95%	55.52% -62.38%		37.13%	33.85% -40.41%
Irritation of eyes and/or throat during cooking	Often (*vs* Rare)	41.45%	64.21%	36.81%	41.27%	37.71%-44.83%		64.51%	61.18%-67.85%		36.18%	32.90%-39.47%
Annual consumption of smoky coal	≥2 tons(*vs* < 2 tons)	20.11%	76.21%	54.36%	20.13%	17.22%-23.03%		76.00%	73.02%-78.98%		54.78%	51.36%-58.21%
Annual consumption of electricity	≥700 kW·h(*vs* < 700 kW·h)	74.92%	47.81%	77.23%	75.38%	72.30%-78.47%		46.98%	43.56%-50.41%		78.09%	75.32%-80.87%
The most frequently used type of stove:fire pit without chimney	Yes(*vs* No)	1.88%	0.38%	1.23%	1.93%	0.92%-2.94%		0.36%	0.00%-0.78%		1.09%	0.41%-1.76%
The most frequently used type of stove:stove in the ground with chimney	Yes (*vs* No)	1.21%	60.79%	21.60%	1.16%	0.40%-1.92%		60.62%	57.20%-64.04%		21.44%	18.62%-24.27%
The most frequently used type of stove:tall stove with chimney	Yes (*vs* No)	25.57%	19.54%	35.09%	25.21%	22.09%-28.33%		19.82%	17.03%-22.61%		35.10%	31.81%-38.39%
The most frequently used type of stove:portable stove	Yes (*vs* No)	6.33%	1.78%	27.73%	6.32%	4.56%-8.08%		1.87%	0.90%-2.84%		27.58%	24.52%-30.64%
^a^: Ways of domestic heating involving smoky coal combustion: coal stove, fire pan, fire pit; Ways of domestic heating not involving smoky coal combustion: no heating, central heating system, electric radiator.												

按年龄或性别分亚组后分析的结果显示，各年龄段的人群或两个性别的人群，各个危险因素暴露率在高、中、低发区基本都围绕在总人群率周围，数值与总人群率的值相差不超过总人群率的1/3。唯一例外的是高发区最常使用地炉的家庭的占比，男性汇报为2.5%，是总人群率（1.2%）的2倍，女性汇报仅为0.2%。并没有某一年龄段或性别的人群表现出危险因素暴露率统一偏高或偏低的现象，各因素的性别、年龄分布都不甚一致。

### 2.3 生活环境方面危险因素暴露情况现在与十年前的比较

表 3中展示了高、中、低发区样本人群整体在现在和十年前，生活环境方面危险因素分别的暴露情况，及其比较结果。

**3:** 调查对象生活环境方面危险因素当前与十年前暴露情况比较 The exposure to living environmental risk factors of the respondents, comparison between nowadays and 10 years ago

	10 years ago			Nowadays		*χ*^2^	*P*
	*n*	%		*n*	%		
High lung cancer area							
Ways of domestic heating involving smoky coal combustion: Yes	571	76.85		396	53.3	90.68	< 0.001
Ventilation or using smoke exhauster during cooking: Yes	256	34.45		389	52.36	48.46	< 0.001
Fume in the kitchen during cooking: Yes	328	44.15		272	36.61	8.77	0.003
Irritation of eyes and/or throat during cooking: Yes	368	49.53		308	41.45	9.77	0.002
Annual consumption of smoky coal: ≥2 tons	382	2.98		149	20.11	170.75	< 0.001
Annual consumption of electricity: ≥700 kW·h	128	21.99		481	74.92	342.09	< 0.001
The most frequently used type of stove is fire pit without chimney: Yes	18	2.42		14	1.88	0.51	0.475
The most frequently used type of stove is stove in the ground with chimney: Yes	58	7.81		9	1.21	37.53	< 0.001
The most frequently used type of stove is tall stove with chimney: Yes	407	54.78		190	25.57	131.85	< 0.001
The most frequently used type of stove is portable stove: Yes	57	7.67		47	6.33	1.03	0.309
Median lung cancer area							
Ways of domestic heating involving smoky coal combustion: Yes	772	97.97		735	93.27	20.75	< 0.001
Ventilation or using smoke exhauster during cooking: Yes	142	18.02		212	26.90	17.85	< 0.001
Fume in the kitchen during cooking: Yes	559	70.94		463	58.76	25.65	< 0.001
Irritation of eyes and/or throat during cooking: Yes	561	71.19		506	64.21	8.78	0.003
Annual consumption of smoky coal: ≥2 tons	626	80.36		599	76.21	3.96	0.046
Annual consumption of electricity: ≥700 kW·h	76	12.60		350	47.81	188.66	< 0.001
The most frequently used type of stove is fire pit without chimney: Yes	50	6.35		3	0.38	43.13	< 0.001
The most frequently used type of stove is stove in the ground with chimney: Yes	466	59.14		479	60.79	0.45	0.504
The most frequently used type of stove is tall stove with chimney: Yes	250	31.73		154	19.54	30.68	< 0.001
The most frequently used type of stove is portable stove: Yes	17	2.16		14	1.78	0.30	0.586
Low lung cancer area							
Ways of domestic heating involving smoky coal combustion: Yes	757	92.88		660	80.98	50.81	< 0.001
Ventilation or using smoke exhauster during cooking: Yes	219	26.87		228	27.98	0.25	0.617
Fume in the kitchen during cooking: Yes	481	59.02		310	38.04	71.82	< 0.001
Irritation of eyes and/or throat during cooking: Yes	462	56.69		300	36.81	64.68	< 0.001
Annual consumption of smoky coal: ≥2 tons	578	71.01		443	54.36	48.27	< 0.001
Annual consumption of electricity: ≥700 kW·h	181	22.82		624	77.23	473.82	< 0.001
The most frequently used type of stove is fire pit without chimney: Yes	145	17.79		10	1.23	129.94	< 0.001
The most frequently used type of stove is stove in the ground with chimney: Yes	155	19.02		176	21.60	1.67	0.196
The most frequently used type of stove is tall stove with chimney: Yes	339	41.60		286	35.09	7.29	0.007
The most frequently used type of stove is portable stove: Yes	285	34.97		226	27.73	9.92	0.002

现在与十年前相比较，高发区以燃煤方式取暖的家庭所占百分比下降幅度最大，由75%-80%降至50%-60%；低发区其次由90%多降至80%；中发区仅略有下降，百分比值保持在90%以上。高发区有抽油烟机或做饭时注意开窗通风的家庭占比由30%多升至50%多；中发区由20%升至接近30%；低发区没有变化，但从十年前起百分比值就也已接近30%。与通风改善相对应，高发区和中发区报告做饭时厨房油烟多、眼咽有刺激感的人的百分比也略有下降；低发区虽厨房通风情况与十年前相当，油烟/眼咽刺激情况却也有所改善。高发区一年使用2吨或更多烟煤的家庭所占百分比显著减小，由50%多降至20%左右；低发区也如是，由70%降至55%左右；中发区仅由80%多略降至75%。与之相反，在三个区内，一年使用700度或更多电的家庭所占百分比都大幅提升，由20%或以下升至50%-80%。在三个区内，日常最常使用无烟囱火塘的家庭所占百分比均已降至2%以下。高发区和中发区最常使用手提炉的家庭占比未变，低发区显著下降；然而从百分比值来看，低发区是30%左右，高、中发区均不足10%。高发区最常使用有烟囱地炉的家庭在十年前就已只占8%，现在更降至不足2%；中发区和低发区占比未变，前者保持在60%，后者保持在20%。高发区最常使用有烟囱高灶的家庭占比由55%大幅降至25%；中发区由30%降至20%，降幅不是最大但目前百分比值最低；低发区由40%降至35%。

分性别或年龄亚组的分析结果未列入表 3中。现在与十年前相比较，男性和女性人群各个危险因素暴露率的变化程度基本都与整体样本人群近似，且在三个区内都是如此。并未出现某危险因素在某一个性别的人群中暴露率变化程度很大，而在另一个性别人群中变化极小；或在某一个性别的人群中暴露率上升而在另一个性别人群中下降的情况。在三个区内都有一些危险因素在高年龄的两个亚组，尤其是60岁-69岁组中，暴露率未变化。

### 2.4 个体危险因素的暴露情况

由于女性基本没有煤矿工作史、吸烟史和饮酒史，这三项危险因素仅报告男性的暴露率。如表 4所示，中发区近50%的男性有煤矿工作经历，是高、低发区的近两倍。而高发区分别有15%和25%的人有其他矿山和涉及烟尘颗粒污染的工种的工作经历，前者是中、低发区的3倍，后者是5倍。饮食习惯方面，高发区60%多的人经常（指每周至少有3天吃，下同）吃蔬菜，中、低发区比例更依次升高至80%。三区都有60%-65%的人经常吃水果。高发区60%的人经常吃猪肉或牛羊肉，比另外两区高出5%-10%；40%的人经常吃鸡鸭鱼虾，是另外两区的两倍。中发区57%的人经常吃豆类或豆制品，比高、低发区略高出5%。高、中发区都有40%-50%的人经常吃蛋类，而低发区不到30%。经常吃奶或奶制品的人偏少，在高发区有27%，在中、低发区更依次降低。高发区40%的人经常吃甜食，是中、低发区的2倍。低发区75%-80%的人经常吃高油高脂肪食物或烟熏制品，中发区降至55%-65%，高发区又降至50%。高、中发区都有75%-80%的男性有吸烟史，而低发区是不到65%。三区都有近30%的男性有饮酒史。高、中发区都有85%-90%的从未吸烟者接触二手烟，而低发区是不到60%。高发区5%的人有肺疾病史，25%的人有家族肿瘤史，虽然数值也不大，但显著高于另外两区。

**4:** 调查对象个体危险因素当前暴露情况 The exposure to individual risk factors of the respondent

Factors		High lung cancer area(*n*=743)	Median lung cancerarea(*n*=788)	Low lungcancer area(*n*=815)	High lung cancer area			Median lung cancer area			Low lung cancer area	
					Standardized rate	95%CI		Standardized rate	95%CI		Standardized rate	95%CI
Coal mining work experience (male only	Yes %(*vs* No)	24.92%	48.91%	29.89%	24.76%	21.64% -27.87%		48.85%	45.40% -52.31%		29.51%	26.39% -32.63%
Other mining work experience	Yes %(*vs* No)	15.07%	4.82%	3.93%	15.12%	12.52%-17.72%		4.66%	3.22%-6.10%		3.97%	2.61%-5.32%
Experience of specific occupation^a^	Yes %(*vs* No)	25.84%	4.57%	5.03%	25.95%	22.78% -29.12%		4.51%	3.07% -5.95%		5.09%	3.56% -6.61%
Days of eating fresh green vegetables (per week, the same below)	3 d-7 d %(*vs* 0-2 d)	58.01%	72.08%	78.90%	58.08%	54.51%-61.65%		72.31%	69.20%-75.43%		78.83%	76.01%-81.64%
Days of eating fruits	3 d-7 d %(*vs* 0-2 d)	61.91%	65.99%	63.56%	62.23%	58.73% -65.73%		66.13%	62.83% -69.43%		63.68%	60.37% -67.00%
Days of eating pork, lamb, beef	3 d-7 d %(*vs* 0-2 d)	59.89%	54.44%	51.90%	60.18%	56.65%-63.71%		54.55%	51.07%-58.03%		52.02%	48.58%-55.47%
Days of eating chicken, duck, fish, shrimp and other aquatic products/seafood	3 d-7 d %(*vs* 0-2 d)	39.84%	18.53%	19.39%	40.04%	36.49%-43.59%		18.35%	15.65%-21.04%		19.41%	16.68%-22.14%
Days of eating bean and bean products	3 d-7 d %(*vs* 0-2 d)	49.93%	56.85%	52.02%	49.93%	46.31%-53.55%		56.86%	53.40%-60.32%		51.82%	48.37%-55.26%
Days of eating eggs	3 d-7 d %(*vs* 0-2 d)	43.74%	49.24%	28.22%	43.82%	40.23%-47.42%		49.13%	45.63%-52.63%		28.28%	25.18%-31.39%
Days of eating milk and dairy products	3 d-7 d %(*vs* 0-2 d)	26.92%	21.83%	17.06%	27.30%	24.06%-30.53%		21.94%	19.04%-24.84%		17.03%	14.43%-19.62%
Days of eating sweets	3 d-7 d %(*vs* 0-2 d)	39.84%	17.01%	22.70%	39.87%	36.32%-43.42%		17.10%	14.46%-19.73%		22.65%	19.77%-25.53%
Days of eating oily and fatty foods	3 d-7 d %(*vs* 0-2 d)	47.11%	53.68%	82.09%	47.23%	43.61%-50.85%		53.77%	50.28%-57.26%		82.35%	79.74%-84.97%
Days of eating pickled or smoked food	3 d-7 d %(*vs* 0-2 d)	51.55%	66.37%	75.83%	51.64%	48.02%-55.26%		66.79%	63.53%-70.04%		75.71%	72.76%-78.67%
History of tobacco use(male only)	Yes %(*vs* No)	75.00%	78.02%	63.22%	75.09%	71.96%-78.22%		78.06%	75.16%-80.96%		63.89%	60.59%-67.20%
Exposure to second hand smoke (never-smokers only)^b^	Yes % (*vs* No)	84.68%	88.49%	58.08%	85.21%	82.73%-87.69%		88.14%	85.87%-90.40%		58.32%	54.92%-61.72%
History of alcohol use (male only)	Yes%(*vs* No)	29.23%	26.78%	28.74%	28.96%	25.68%-32.24%		27.07%	23.96%-30.18%		28.45%	25.43%-31.48%
History of respiratory diseases except for lung cancer^c^	Yes% (vs No)	4.98%	0.38%	2.09%	4.64%	3.19%-6.10%		0.37%	0.00% -0.80%		1.90%	1.00% -2.81%
Family history of cancer^d^	Yes %(*vs* No)	24.63%	5.20%	10.67%	24.66%	21.55%-27.77%		5.12%	3.59%-6.64%		10.91%	8.75%-13.07%
^a^: Specific occupation included work experience in metallurgy, chemical industry, construction, machinery manufacturing, textile printing and dyeing, papermaking, tanning, cooking; ^b^: There were 1, 563 never-smokers in the 2, 346 respondents, 1, 352 of which had information about second hand smoke exposure; ^c^: The options in the questionnaire included tuberculosis, COPD, asthma, silicosis or pneumoconiosis, interstitial lung disease and pulmonary sarcoidosis, other respiratory diseases; ^d^: Including first (parents, children, siblings), second (grandparents, grandchildren, uncles and aunts) and third (cousins) degree relatives. COPD: chronic obstructive pulmonary disease.												

除上述煤矿工作史、吸烟史和饮酒史有明显性别差异外，与环境危险因素类似，各年龄段的人群或两个性别的人群，个体危险因素暴露率在高、中、低发区基本都围绕在总人群率周围，个别与总人群率的值相差略大，但也不超过其1/2。并没有某一年龄段或性别的人群表现出危险因素暴露率统一偏高或偏低的现象，各因素的性别、年龄分布都不甚一致。

## 3 讨论

### 3.1 高发区和中发区的肺癌危险因素

1990年-2013年，宣威作为一个整体，肺癌死亡率始终是全国农村水平的数倍；宣威市内肺癌的高、中、低发区分布维持稳定^[7]^。

根据我们本次调查数据分析显示各个因素的分布情况，我们认为高发区当前首要的肺癌危险因素很可能已经从1970年代的烟煤燃烧转为了烟草使用和二手烟暴露。在1970年代，由于在室内采用无烟囱火塘燃烧烟煤带来的室内空气污染（含致癌物质）这一问题十分严峻，研究者们都未将烟草使用列为宣威地区肺癌高发的主要危险因素^[8]^。然而，早在1950年代，就已有流行病学研究^[9-12]^证实了吸烟是导致肺癌首要的危险因素。在当前高发区烟煤用量整体锐减的情况下，烟草流行的效应可能凸显出来。本课题组比较1990年-1992年、2004年-2005年、2011年-2013年三个时间段的男女肺癌死亡率，发现男女肺癌死亡率间的差距在变大，这可能是烟草为当前肺癌主要危险因素的一个佐证。另外，高发区在非煤矿山工作过的人数，从事过建筑等有职业烟尘暴露的工种的人数都多于另外两区。前期研究已表明，煤矿工人、锡矿工人是肺癌发生的高危人群^[13-15]^。第三，高发区比中、低发区有更多人有肺疾病史和家族肿瘤史。过去有研究^[16]^证实过肺结核和肺部慢性炎症的发生可以在后续多年内增加患者患肺癌的风险。而基因型可能通过增加机体对空气中燃煤释放污染物的敏感性而增加肺癌风险^[17]^。但是，高发区有肺疾病史和家族肿瘤史的人所占百分比数值都很不高，它们的效应有无和效应强度还需要进一步的定性/定量研究。第四，在饮食习惯方面，高发区居民吃蔬菜相对较少，吃甜食相对较多。关于蔬菜与肺癌发生相关性的前期研究结果并不完全一致。有研究^[18]^认为，经常食用新鲜蔬菜水果可降低肺癌风险，但在宣威地区开展的另一项研究结果^[19]^显示，食用绿色蔬菜是肺癌的危险因素，因为其表面积大，接触吸收空气中的多环芳烃等物质的能力强。

1990年-2005年，有些中发区乡镇肺癌的标化死亡率显著上升，其中就包括格宜镇，上升2倍以上（虽然2005年-2013年其肺癌死亡率又下降了40%）^[7]^。本次调查的数据显示，开采和使用烟煤很可能是目前中发区重要的肺癌危险因素。中发区从十年前到现在，都有近80%的家庭年均使用烟煤2吨或以上，超过90%的家庭以燃煤方式取暖。另外中发区居民较不注意厨房的通风，使得很多人家做饭时油烟很重。中发区有煤矿工作经历的男性人数是另两区的二倍。在个人生活习惯方面，中发区居民吸烟（男性）和二手烟暴露的情况也很严峻，与高发区无异。中发区有60%的人家中最常使用有烟囱地炉，远高于另外两区。在改炉改灶工程废弃无烟囱火塘，改用有烟囱的炉灶后，还没有后续研究探讨这些炉灶和肺癌风险间的相关性，这可以作为未来研究的方向之一。

### 3.2 低发区应注意的肺癌危险因素

低发区当前有75%-80%的人经常吃高油高脂肪食物或烟熏制品，而常吃肉、鱼、蛋、豆、奶等食品的人较少。既往有研究提出，高脂饮食、腌制熏制食品是肺癌的危险因素，常吃蛋类、肉类、鱼类、禽类、海产品、奶制品可以减少患肺癌的风险^[20]^。另外，低发区居民也较不注意厨房的通风。低发区65%的男性有吸烟史，60%的从未吸烟者接触二手烟暴露，55%的家庭每年使用2吨或更多的烟煤。在宣威市内部比较时，烟草和烟煤在低发区的暴露率水平低于高和/或中发区，但它们也不应在低发区居民中被忽视。

### 3.3 对宣威地区肺癌死亡率未下降原因的探讨

截止到当前为止，宣威地区的肺癌高、中、低发区分布始终保持未变。其原因有可能是：第一，危险因素暴露的蓄积效应。从改炉改灶结束到现在，虽然高发区烟煤用量已显著下降，大部分居民也使用电炉，但可能由于蓄积效应，肺癌死亡率下降的拐点还未出现。Peto等^[21]^曾报告过由于烟草的累积作用，吸烟者戒烟后患肺癌风险虽然下降但依然高于非吸烟者，同理我们推测室内空气污染对肺癌的影响也存在类似的累积效应。未来若持续监测宣威肺癌死亡率，可能会发现高发区肺癌死亡水平下降，若配合观测青年人群肺癌患病率，可进一步明确近期宣威肺癌的危险因素。第二，干预措施的作用未发挥到位。目前在中、低发区仍有半数至75%的居民家中使用较多烟煤。另外，除火塘外，其他炉灶（尤其是地炉）的安装情况和使用过程中烟雾排放情况目前是未知的。还有现在与十年前比较时，可见老年人群中，燃煤取暖、厨房油烟等多个环境危险因素暴露率未变化。第三，出现新的肺癌危险因素。例如上述的烟草流行和二手烟暴露。另外，从本次调查的数据中我们可以看出，目前宣威地区用电量较过去激增，这必然带来发电量的大幅增加。宣威地区火电厂采用燃煤发电，因此火电厂用煤量的上升可能带来室外环境大气的污染，导致宣威居民虽不再受到室内空气污染的暴露，但转为受到室外环境大气污染的暴露。

### 3.4 对宣威地区未来研究方向的建议

基于本次调查的数据和上述讨论分析，我们建议：第一，持续监测宣威地区居民的肺癌死亡率和患病率，观察其趋势，观察肺癌水平的拐点。第二，继续研究烟煤使用、烟草流行（包括二手烟）和环境空气污染与肺癌间的相关性，并探索烟草烟雾和煤炭燃烧烟雾的交互作用。2014年的一项研究^[22]^证明，烟煤使用可以加强吸烟对宣威男性造成的患肺癌风险。第三，观察研究目前与肺癌相关性尚无定论的因素，包括饮食习惯和炉灶类型。第四，矿山或其他例如建筑等职业人群，可以作为一类特殊人群纳入研究。本次调查的数据显示，这类人群在吸烟、饮酒、使用炉灶类型等方面与普通人群有一定差异性，职业暴露和个人生活方式相关危险因素应综合考虑。

### 3.5 研究的局限性

本次调查设计为分层随机抽样，但数据显示样本人群的年龄结构在三个区之间有统计学差异，这可能与区域的地形和住户的分布有关。另外，调查采用回忆法，不能排除回忆偏倚，特别可能影响对十年前使用量、（戒烟者）曾经吸烟量等问题的回答情况。

## 4 结论

烟草流行包括二手烟暴露、烟煤使用、职业暴露可能是当前宣威肺癌仍旧高发的原因。炉灶类型、饮食习惯和环境大气污染与宣威肺癌间的相关性值得进一步研究。此外，烟草烟雾与烟煤烟雾间的交互作用也需要进一步研究。未来宣威肺癌水平可能出现下降拐点，高、中、低发区的分布也可能发生变化。
